# Social Context and Reproductive Potential Affect Worker Reproductive Decisions in a Eusocial Insect

**DOI:** 10.1371/journal.pone.0052217

**Published:** 2012-12-14

**Authors:** Boris Yagound, Pierre Blacher, Stéphane Chameron, Nicolas Châline

**Affiliations:** Laboratoire d'Ethologie Expérimentale et Comparée, E.A. 4443, Université Paris 13, Sorbonne Paris Cité, Villetaneuse, France; University of Sheffield, United Kingdom

## Abstract

Context-dependent decision-making conditions individual plasticity and is an integrant part of alternative reproductive strategies. In eusocial Hymenoptera (ants, bees and wasps), the discovery of worker reproductive parasitism recently challenged the view of workers as a homogeneous collective entity and stressed the need to consider them as autonomous units capable of elaborate choices which influence their fitness returns. The reproductive decisions of individual workers thus need to be investigated and taken into account to understand the regulation of reproduction in insect societies. However, we know virtually nothing about the proximate mechanisms at the basis of worker reproductive decisions. Here, we test the hypothesis that the capacity of workers to reproduce in foreign colonies lies in their ability to react differently according to the colonial context and whether this reaction is influenced by a particular internal state. Using the bumble bee *Bombus terrestris*, we show that workers exhibit an extremely high reproductive plasticity which is conditioned by the social context they experience. Fertile workers reintroduced into their mother colony reverted to sterility, as expected. On the contrary, a high level of ovary activity persisted in fertile workers introduced into a foreign nest, and this despite more frequent direct contacts with the queen and the brood than control workers. Foreign workers' reproductive decisions were not affected by the resident queen, their level of fertility being similar whether or not the queen was removed from the host colony. Workers' physiological state at the time of introduction is also of crucial importance, since infertile workers failed to develop a reproductive phenotype in a foreign nest. Therefore, both internal and environmental factors appear to condition individual reproductive strategies in this species, suggesting that more complex decision-making mechanisms are involved in the regulation of worker reproduction than previously thought.

## Introduction

The decision of where and when to reproduce has a crucial impact on an individual's fitness. In eusocial Hymenoptera workers normally have very few opportunities to lay eggs in their mother colony, either because of a self-restraint deriving from their inclusive fitness interests, or because of constraint mechanisms that coercively prevent their reproduction [1]. Worker reproductive parasitism, i.e., when a worker leaves its native nest, enters an unrelated colony and reproduces, is a powerful alternative strategy enabling workers to increase their direct fitness [2]. This possibly widespread reproductive strategy [3] has currently been described in several species ranging from honey bees [4]–[9], sweat bees [10], [11], stingless bees [12] and bumble bees [13]–[15], to vespine wasps [16] (Table 1). The types and rates of parasitism vary according to species, but a common characteristic of this phenomenon is that intraspecific parasite workers always reproduce to a significant extent at the expense of host colonies (Table 1). Reproductive options available to workers are thus much more varied than traditionally considered [2]. Despite the potential spread of this phenomenon, the proximate mechanisms at the basis of such worker reproductive strategies remain largely unknown, and investigating them is therefore of paramount importance to understand what ultimately shapes reproductive decisions in eusocial insects.

**Table 1:** Worker reproductive parasitism in eusocial species.

Eusocial species	Type of WRP	Rate of WRP^a^	Reference
**Honey bees**			
*Apis cerana*	Intercolony parasitism in hopelessly queenless colonies	5.5%	[8]
*Apis florea*	Intercolony parasitism in hopelessly queenless colonies	22.5%	[6]
*Apis mellifera*	Intercolony parasitism in hopelessly queenless colonies	7.7%	[9]
*Apis mellifera capensis*	Intercolony parasitism in queenright colonies	6.4%^b^	[7]
**Sweat bees**			
*Lasioglossum malachurum*	Intercolony parasitism in queenright colonies	?^c^	[10]
**Stingless bees**			
*Melipona scutellaris*	Intergenerational parasitism in queenright colonies	18.5%	[12]
**Bumble bees**			
*Bombus deuteronymus*	Intercolony parasitism in queenright colonies	6.7%	[15]
*Bombus occidentalis*	Intercolony parasitism in queenright colonies	?^d^	[13]
*Bombus terrestris*	Intercolony parasitism in queenright colonies	2.1%	[14]
**Vespine wasps**			
*Vespula consobrina*	Interspecific parasitism in *Vespula atropilosa* colonies	?	[16]

WRP, worker reproductive parasitism; ?, unknown.

a In terms of proportion of parasite-worker-derived males.

b These are queens and not males due to thelytokous parthenogenesis [7]. Parasite workers also invade colonies of the subspecies *Apis mellifera scutellata*, causing the death of the host colonies [4], [5].

c These are queens and not males due to worker mating [10].

d WRP has not yet been formally shown in this species but is likely to occur [13].

Here we test whether workers of the annual bumble bee *Bombus terrestris* adjust their ovarian activity according to their social environment. *B. terrestris* is a good model system given the unusual possibility for workers to directly increase their fitness in two ways, namely through intercolony parasitism [14] (Table 1) and direct competition with their nestmates –the queen and the other workers– at the end of the colony cycle [17]–[19]. Self-restriction is thought to explain the levels of worker reproduction in this species during the early stages of the colony cycle [20]–[22] where workers with activated ovaries refrain from reproducing [23]. A subsequent change in a still unidentified queen signal linked with the production of young queens appears to trigger the onset of overt intracolonial competition [20], [24]–[26] (but see [27]). Prior to this so-called competition phase (see Materials and Methods, “Study Organism”), potentially competing workers have reduced ovary activation, with however a marked heterogeneity due to dominance interactions [19], [23], [28]. Since parasite workers in unrelated host colonies have no risks of jeopardizing their inclusive fitness, they would benefit from reproducing before the host workers, thus enhancing their direct fitness [14]. One hypothesis allowing to explain worker reproductive advantage in foreign nests is a non-responsiveness to the resident queen signal, but to our knowledge this has never been investigated. In the present study, we assessed the role of the social environment (i.e., native vs. foreign colony) on worker reproductive decisions and examined whether the presence of the queen has a specific influence in this process. We expected that fertile workers reintroduced into their native colony would decrease their fertility [21]. Conversely, the inclusive fitness theory predicts that fertile bees introduced into a foreign nest would maintain their fertility, favouring in this case direct fitness benefits at no relatedness cost. Based on the existing differences in ovary activation between workers of the same colony (see above), we tested whether workers' initial fertility before entering a foreign nest is a key factor in explaining worker reproduction in unrelated colonies. Finally, as workers have to be in direct contact with the queen to perceive her signal [25], we examined whether an absence of ovarian inhibition could be due to a behavioural avoidance of the queen.

## Materials and Methods

### Study Organism

The buff-tailed bumble bee *B. terrestris* form annual colonies headed by a single and singly mated queen [29], [30], and characterized at the end of the colony cycle by a competition phase during which the queen and the reproductive workers compete with each other over male production by means of overt aggressions and egg-eating [17]–[19], [25]. Colonies were obtained from GTICO SARL (Villeneuve l'Archevêque, France) a few days after the emergence of the first worker. Each experimental colony was before the competition phase and had a queen, brood at every developmental stage and was restricted to 20 workers in order to control the possible effects of workers' density. Colonies were reared in wooden boxes (17.5×26×15 cm) in a dark room at a temperature of 30±2°C and a relative humidity of 55±5%. They were fed *ad libitum* with sugar syrup and fresh pollen. Daily observations of the colonies were performed under a low red light through a glass placed above the box, allowing the localization of new emergences and the detection of the initiation of the competition phase (at least one of these characteristics: two egg-cells or more opened for at least two consecutive days, worker oviposition, egg-eating, clear signs of cell destruction [17], [19]). Husbandry and experimental procedures used in this study fulfilled all the legal requirements concerning insect experimentation of France.

### Experimental Design

**Figure 1:**
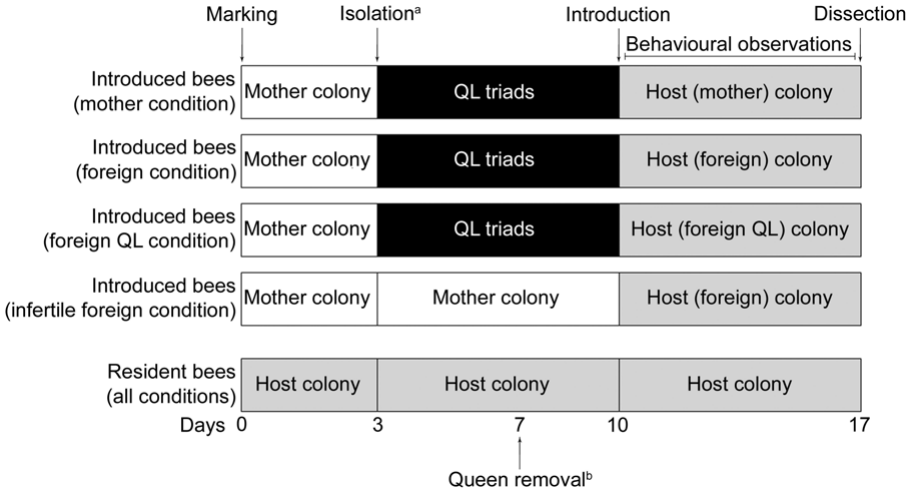
Experimental design showing the consecutive groups that the bees encountered in the various conditions. QL triads consisted of one focal bee and two one-day-old foreign bees. Behavioural observations occurred between days 10 and 17. QL, queenless. ^a^Isolation did not concern introduced bees in the infertile foreign condition or resident bees in all conditions. ^b^Queen removal in the host colony was performed in the foreign QL condition only.

The experimental setup is based on the protocol of Alaux et al. [21]. Experiments lasted 17 days and consisted in introducing groups of five fertile or infertile workers into colonies of various social structures, namely their native colony or a foreign colony with or without the queen (Figure 1), at a time in the colony cycle where normally no workers reproduce (i.e., before the competition phase). In the mother condition (*n* = 6 colonies), 10 newly emerged workers originating from the same colony were labelled with numbered tags (Opalith Plättchen, Friedrich Wienold, Germany) glued in their thorax, and were reintroduced into their native colony. Five of the marked bees (hereafter referred to as “resident bees”) were used as controls and stayed in their colony during all the experiment. The other five ones stayed three days in their colony so that they could learn their colonial odour. After this period, they were isolated and individually placed into queenless triads with two one-day-old callow workers originating from different colonies, and fresh pupae in order to stimulate laying. They stayed in these triads during seven days, which is the necessary duration for the ovaries to fully develop [23]. In these conditions, the oldest bee is always the laying-one after the seventh day [21]. The five marked laying bees were then reintroduced into their mother colony during seven days (“introduced bees”). The experimental design guaranteed that nothing differed between the various conditions except the social environment the introduced bees encountered. In the foreign condition (*n* = 6 colonies), the protocol was identical, but this time the five introduced bees were introduced into a foreign host colony. All introduced bees came from a different colony, and callow workers in queenless triads also came from different colonies, so that any habituation process was prevented. The five resident bees were taken from the foreign host colony. In order to determine more precisely the influence of the queen on foreign workers, we created another condition (foreign queenless condition, *n* = 6 colonies) where the introduced bees were placed in a foreign colony where the queen has been removed three days prior to the introduction, which allowed the resident bees to start laying. We finally wanted to assess the influence of the workers' fertility state on their reproductive decisions (infertile foreign condition, *n* = 6 colonies). To this purpose the introduced bees did not develop their ovaries in queenless triads, but instead stayed 10 days in their mother colony before being introduced into a foreign colony with a queen (Figure 1).

### Behavioural Observations

In order to measure the behaviour of the introduced bees, each colony with a queen (*n* = 18 colonies) was observed 30 min twice a day, morning and afternoon, during a one-week period. During these observations, scan sampling of the 10 focal workers (five introduced + five resident bees in each observed colony) was performed every 2 min during 15 min. The occurrence of all behavioural acts linked to worker tasks was then recorded: collecting food (collecting pollen or sugar syrup), patrolling outside the brood, working on honey pots (manipulating the wax or inserting food inside), inspecting the brood (moving on the brood with repeated antennal contacts), brood care (feeding larvae, manipulating the wax of larvae cells, or incubating pupae), and inactivity (immobility during several seconds) (see [31]). The localization of the bees in the nest, namely inside or outside the brood, was recorded at each scan. Because antennating the queen is probably the best way for the workers to evaluate her putative signal [20], [21], [25], [26], we also recorded the occurrence of the antennal contacts between the 10 focal workers and the queen during 15 min.

### Fertility Measurement

At the end of the observation period, i.e., seven days after the bees' introduction, the 10 focal workers of each colony (*n* = 24 colonies) were dissected and the mean size of the eight terminal oocytes was taken as a measure of the ovarian development. Workers establish a dominance hierarchy in the colonies and exhibit different ovarian developments [19], [23], [28], hence the response of the different introduced or resident bees were not independent observations. We thus took the mean ovarian development of all the introduced or resident bees of a colony, in order to have one observation per condition per colony. A classification of the workers was performed with respect to their ovarian development [23], depending on the presence of non-developed (mean size of the terminal oocytes ≤1.18 mm) or developed ovaries (mean size of the terminal oocytes >1.18 mm). This allowed estimating the mean percentage of workers with developed ovaries in each condition. In parallel, control bees with fully developed or non-developed ovaries were used to assess the dynamics of workers' ovarian activity. Fertile control bees (*n* = 6 groups) consisted of triads of workers created in the same conditions as above, but this time the bees stayed together during 14 days to control the possible effects of age on ovarian development in a queenless situation. This control represented the *a priori* maximal expected value for the ovarian development in the experimental groups. Conversely, infertile control bees (*n* = 6 colonies) consisted of non-manipulated colonies before the competition phase, of whom five bees of identical age were dissected 17 days after their emergence, representing an *a priori* minimal value for the ovarian development.

### Statistical Analyses

Ovarian development of groups of five introduced or resident bees, percentage of bees classified as having developed ovaries, percentage of scans the bees spent on the brood, percentage of scans they spent in all behavioural tasks recorded, and rate of antennal contact with the queen (per scan per bee) were compared between conditions using one-way ANOVAs with the Monte Carlo procedure [32], followed by post-hoc exact permutation tests corrected for multiple comparisons with the Bonferroni sequential method. Comparisons between resident and introduced bees for all these categories, and between the first and the last day of the observation period were performed by exact permutation tests. Correlations between ovarian development and rate of antennal contact with the queen (per scan per bee), and between the percentage of scans bees spent on the brood and the percentage of scans they spent caring for the brood were carried out with Pearson's test with the Monte Carlo procedure. All statistical analyses were performed with StatXact 8.0 software (Cytel Software Corporation, Cambridge, United States). Statistical significance was set at *p*<0.05.

## Results and Discussion

**Figure 2:**
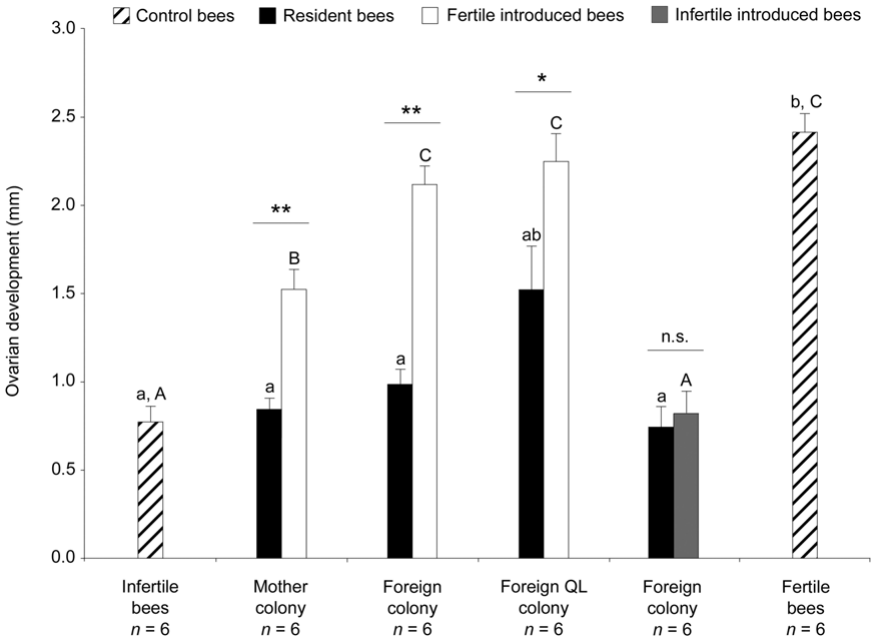
Ovarian development of control, resident and introduced bees in the various conditions. Groups of five fertile or infertile bees were introduced into their mother colony or a foreign colony containing or lacking the queen. Resident bees were native workers from the host colony. Infertile control bees were randomly taken from non-manipulated colonies before the competition phase (see Materials and Methods). Fertile control bees were laying workers taken from triads of isolated workers. All workers were of the same age. The different letters denote statistical differences; each analysis (one-way ANOVA with the Monte Carlo procedure followed by post-hoc exact permutation tests corrected for multiple comparisons with the Bonferroni sequential method) is represented by uppercase or lowercase letters. Comparisons between resident and introduced bees for each condition were performed by exact permutation tests. QL, queenless; n.s., not significant; * *p*<0.05; ** *p*<0.01. Data are represented as mean ± standard error.

Ovarian development of introduced bees was affected by the social environment they encountered (one-way ANOVA, *F*_5,30_ = 38.9, *p*<0.0001; Figure 2). After one week, fertile bees introduced into a foreign colony showed a significantly higher ovarian activity than those reintroduced into their mother colony (exact permutation test: *p* = 0.002; Figure 2); this fertility level did not statistically differ from the ovarian activity of fertile control bees (*p* = 0.32; Figure 2). Eighty-four percent of the fertile bees introduced into a foreign nest (*n* = 6 colonies) had developed ovaries seven days later, whereas only 55.6% (*n* = 6 colonies, *p* = 0.037) did when reintroduced into their native nest. Social context therefore appears to dramatically influence fertile workers reproductive decisions, resulting in a fine tuning of their ovarian activity pattern to their fitness interests. In order to assess if the host queen still had an influence on foreign workers' fertility, we introduced fertile bees in six other foreign queenless nests. One week later, their ovarian activity was similar to that observed both in the foreign condition where the queen was present and in reproductive control workers (both *p* = 1; Figure 2). As foreign workers' fertility was not affected by the presence of a queen in the host colony, fertility differences in bees introduced into their mother compared to a foreign nest are likely due to differences in queen signal influence, suggesting that foreign workers do not respond to the host queen signal. Whether or not this signal differs between colonies is to be determined but queen swapping experiments tend to support a species-specific signal [26], which would be expected if it functions as an honest signal [33]. We therefore hypothesize that other cues –probably of a chemical nature– perceived by workers in the host nest are involved in the decision-making process.

Parasite workers probably have a short window of opportunity to lay their eggs in a host colony [20]. Their physiological state, or reproductive potential, at the time of joining might therefore be of crucial importance in influencing their reproductive success, even more so that resident workers also will compete for reproduction in the late stages of the colony cycle. To examine this proximate factor, we introduced infertile workers into six foreign nests with a queen. One week later, their ovarian activity was similar to that of sterile control bees (exact permutation test: *p* = 1; Figure 2), and significantly smaller than the fertility of all other introduced and control groups (all *p*<0.039). This means that these workers never developed their ovaries despite being in a foreign colony. Drifting, i.e., the movement of workers between nests [34], is a common trait in bee species [3], and is often characterized by the presence of non-reproductive workers in foreign nests, likely due to orientation errors (e.g., [4], [8], [9], [11], [14]). Since foreign workers' kin-selected interests do not vary with their fertility state, our results rather suggest that pre-developed ovaries constitute a necessary condition for some workers to acquire a parasitic phenotype and that initial fertility might also represent a key parameter that promotes their departure from the mother colony. This situation is in fact likely to occur in natural conditions, since a high proportion of workers can have developed ovaries during the social phase [19], [23], [28], and it has actually been shown in semi-natural conditions that workers can parasitize host colonies before the competition point [14]. Studies in the field are however, to our knowledge, still lacking. Ecological data in wild *B. terrestris* colonies (e.g., proportion of drifter workers according to the phase of the colony cycle, degree of synchronization between nests, and population structure) would be useful to deepen our understanding of worker reproductive parasitism and drifting in this species.

In resident bees, ovarian development was significantly smaller than the development of fertile control bees, and not significantly different from those of infertile control bees in all but the foreign queenless condition (exact permutation tests: all *p*<0.031 and all *p*>0.65 respectively; Figure 2), where they showed intermediate levels of ovarian activity, lying between fertile and infertile bees (both *p*>0.12). This confirms the known influence of the mother queen on workers' ovarian activity [20], [21], [23], the latter increasing once the colony is made queenless. These results taken together also show that the introduction of fertile bees has no effect on the ovarian activity of resident bees. When infertile bees were introduced into a foreign colony, resident and introduced bees exhibited a very low and similar ovarian activity (*p* = 0.66; Figure 2). In all other conditions, fertile introduced bees exhibited a higher ovarian activity than resident bees (mother condition: *p* = 0.002; foreign condition: *p* = 0.002; foreign queenless condition: *p* = 0.039). This shows that the ovarian regression of the fertile bees reintroduced into their mother colony is not complete after seven days.

Our results show that the host queen has no effect on foreign workers' reproductive status. The proximate factors underlying this absence of foreign workers' sensitivity could be diverse. Queen signal is supposed to be a non-volatile secretion [25], which is probably transmitted by the queen herself, and also through the wax surrounding the brood on which she is constantly present. Therefore, foreign workers could just behaviourally reduce or avoid contacts with the queen signal, as is the case in the Cape honey bee *Apis mellifera capensis* [35]. To tackle this question, we assessed the behaviour of all introduced and resident bees in all conditions where the queen was kept in the colony (Tables 2, 3 and 4). Here we focused on behaviour linked to the perception of the putative queen signal, namely the antennal contacts with the queen, brood care and the time the individuals spent on the brood. Except for the antennation with the queen, the behaviour of introduced bees differed significantly between the various conditions (brood care: one-way ANOVA, *F*_2,81_ = 4.06, *p* = 0.021; presence on the brood: *F*_2,81_ = 8.53, *p* = 0.0004). When introduced into a foreign colony, both fertile and infertile bees performed more brood care and were more often located on the brood than the resident bees (exact permutation tests: all *p*<0.009; Tables 3 and 4), with the two variables highly correlated (Pearson's correlation, foreign condition: *r* = 0.67, *p*<0.0001; infertile foreign condition: *r* = 0.86, *p*<0.0001). By contrast, fertile bees reintroduced into their native nest did not differ from resident bees (all *p*>0.06; Table 2). In addition, fertile bees were significantly less often located on the brood and performed less brood care when introduced into their mother compared to a foreign colony (*p* = 0.001 and *p* = 0.033 respectively). Introduced bees in the various conditions did not differ in their rate of antennal contact with the queen (*F*_2,81_ = 1.97, *p* = 0.15). The queen seemed to be highly attractive for the introduced bees since they performed more antennations on her than the resident bees (all *p*≤0.05; Tables 2, 3 and 4). These results were even more pronounced in fertile foreign bees (Table 3); their ovarian development was positively correlated with their rate of antennal contact with the queen (*r* = 0.47, *p* = 0.018). Interestingly, fertile foreign bees thus exhibit striking similarities with elite workers, i.e., intra-colonial workers that stay close to the queen and are the first to reproduce at the onset of the competition phase [28]. Furthermore, this attractiveness for the queen was consistent over time, since introduced bees did not differ in their rate of antennation with the queen along the observation period (first vs. last day, mother condition: *p* = 0.52; foreign condition: *p* = 0.47; infertile foreign condition: *p* = 0.70). Contrary to the queen signal avoidance prediction, fertile bees introduced into a foreign colony thus performed more brood-related tasks and more antennal contacts with the queen than the resident bees (all *p*<0.009; Table 3). Workers were therefore extensively exposed to the foreign queen signal but did not respond to it, while they reverted to sterility when exposed to their mother signal. In a more general way, this stresses the fact that workers base their reproductive decisions on more complex cues than previously considered, and that this plasticity allows them to maximize their fitness interests [20].

**Table 2:** Behaviour and localization of the bees in the mother condition.

	Resident bees, *n* = 30	Introduced bees, *n* = 29	*p*
**Antennal contacts with the queen**	0.87±0.09	1.33±0.21	0.045
**Collecting food**	7.75±1.60	6.43±1.09	0.55
**Patrolling outside the brood**	39.43±3.60	27.29±3.74	0.022
**Inactivity**	9.72±2.43	16.02±2.94	0.07
**Working on honey pots**	1.73±0.37	4.06±0.59	0.001
**Inspecting the brood**	23.99±2.50	20.87±2.04	0.34
**Brood care**	16.43±2.37	23.83±2.95	0.06
**Presence on the brood**	42.53±4.36	49.67±5.03	0.29

Rate of antennation with the queen (per scan per bee), task allocation (percentage of scans) for all behavioural tasks recorded, and presence on the brood (percentage of scans) for resident and introduced bees in the mother condition. Rare activities (representing <1% of total acts) were excluded from the analysis (exact permutation tests). Data are presented as mean ± standard error.

**Table 3:** Behaviour and localization of the bees in the foreign condition.

	Resident bees, *n* = 30	Introduced bees, *n* = 28	*p*
**Antennal contacts with the queen**	1.06±0.14	2.01±0.30	0.003
**Collecting food**	6.61±0.75	9.36±1.35	0.08
**Patrolling outside the brood**	30.81±3.23	12.85±1.89	<0.0001
**Inactivity**	5.48±1.11	4.60±1.66	0.67
**Working on honey pots**	1.88±0.41	4.12±0.62	0.003
**Inspecting the brood**	28.05±2.14	32.14±1.53	0.13
**Brood care**	24.89±2.39	33.76±2.22	0.009
**Presence on the brood**	55.19±3.48	72.02±3.04	0.001

Rate of antennation with the queen (per scan per bee), task allocation (percentage of scans) for all behavioural tasks recorded, and presence on the brood (percentage of scans) for resident and introduced bees in the foreign condition. Rare activities (representing <1% of total acts) were excluded from the analysis (exact permutation tests). Data are presented as mean ± standard error.

**Table 4:** Behaviour and localization of the bees in the infertile foreign condition.

	Resident bees, *n* = 30	Introduced bees, *n* = 27	*p*
**Antennal contacts with the queen**	1.09±0.13	1.59±0.22	0.050
**Collecting food**	7.07±1.31	11.23±1.15	0.023
**Patrolling outside the brood**	43.71±3.26	22.96±2.05	<0.0001
**Inactivity**	3.34±1.07	5.99±1.53	0.16
**Working on honey pots**	1.26±0.24	2.17±0.41	0.053
**Inspecting the brood**	25.86±1.47	25.89±1.72	0.99
**Brood care**	17.01±2.05	29.55±2.17	<0.0001
**Presence on the brood**	44.13±2.95	57.24±3.22	0.004

Rate of antennation with the queen (per scan per bee), task allocation (percentage of scans) for all behavioural tasks recorded, and presence on the brood (percentage of scans) for resident and introduced bees in the infertile foreign condition. Rare activities (representing <1% of total acts) were excluded from the analysis (exact permutation tests). Data are presented as mean ± standard error.

Worker reproductive parasitism is always described as costly for host colonies as it is usually associated with an absence of work from parasite workers [4], [5], [14], [16], [35]. In our study however, *B. terrestris* workers performed more brood-related tasks in a foreign colony than in their native nest. Fertile individuals have already been reported to be more inclined to attend the brood than infertile ones [28], [36], [37]. In the context of intraspecific parasitism, their high rates of brood care may functionally be seen as an investment in the host colony, or a concession from foreign workers to guarantee acceptance [38], [39]. Although these results have to be confirmed in natural conditions and supplementary investigations are required, this indicates that host colonies may actually benefit from welcoming in-nest worker intruders. Costs and benefits linked to the presence of foreign workers should thus be carefully weighed out not only for the intruders themselves but also for the host colonies in order to properly characterize this intraspecific relationship. In the absence of indirect fitness benefits, reproductive skew theory and transactional models [39] need to be explored in order to understand how each party (i.e., foreign workers, resident workers and the queen) can influence each other's behaviour and reproductive choices. A balanced or even a favourable costs-benefits outcome of accepting foreign workers could explain the so far unresolved high observed tolerance of bee colonies towards intruders [2].

## Conclusions

This study investigated the proximate factors affecting worker reproductive decisions. We show that workers are even more plastic than previously thought [1], [40], [41]. This plasticity appears to be a key factor in their ability to adjust their fertility according to the social context they encounter, in line with their fitness interests and as a result of complex information processing of multiple cues. Decision-making in *B. terrestris* workers depends on trade-offs between indirect fitness costs and direct fitness benefits. When workers are in their native colony, self-restraint is observed until the queen signal changes, leading to intracolonial competition [20], [25]. In a foreign nest, the absence of immediate inclusive fitness costs and the possibility of high direct fitness benefits give no reason to restrain from reproducing, and open a window of opportunity for worker reproductive parasitism. In eusocial insects, because most of the time they do not directly participate in reproductive activities [40], [41], workers have long been characterized as having very few reproductive options, their behaviour being determined by factors such as relatedness, colony efficiency or competition among reproductives [1], [22], [42]–[45]. In fact, worker reproductive options are many and diversified [2], and in this context individual decision-making is a key component of the reproductive behaviour of workers both inside and outside their society. This plasticity is also likely to ultimately shape patterns of reproductive skew in species as a whole through a fine balance between colony-level and individual selection.
